# Mathematical Modeling for Hepatitis B Virus: Would Spatial Effects Play a Role and How to Model It?

**DOI:** 10.3389/fphys.2020.00146

**Published:** 2020-02-25

**Authors:** Shawn Means, Md A. Ali, Harvey Ho, Jane Heffernan

**Affiliations:** ^1^School of Natural and Computational Sciences, Massey University, Auckland, New Zealand; ^2^Department of Mathematics and Statistics, York University, Toronto, ON, Canada; ^3^Auckland Bioengineering Institute, The University of Auckland, Auckland, New Zealand

**Keywords:** hepatitis B virus (HBV), liver, mathematical model, metabolic zone, NTCP

## Introduction

For nearly 250 million people worldwide suffering from chronic hepatitis B virus (HBV) infection, there are few effective options for treatment (Schweitzer et al., 2015). Elimination of HBV from liver tissue remains an elusive goal; chronic HBV is exceptionally difficult to treat. It results mainly from maternal-neonatal vertical infection but also in roughly 5% of horizontal infections (Rehermann, 2013), and is characterized by the entrenchment of viral covalently closed circular DNA (cccDNA) in host nuclei. cccDNA persistence and HBV resurgence under multiple scenarios [e.g., immunosuppression for chemotherapy (Hoofnagle, 2009)] are associated with liver pathologies such as tissue damage (fibrosis, cirrhosis) and hepatic carcinomas (Ikeda et al., 2010). Current treatments are aimed at mitigating such long-term pathological effects and require sustained and prolonged protocols, yet do not eradicate cccDNA leading to reactivation upon cessation (Guo and Guo, 2015). cccDNA entrenchment further evades detection—with some occult DNA levels well under 100 copies/ml—complicating transfusions or transplantations (Schmeltzer and Sherman, 2010).

Efforts at mimicking and exploiting the successful immune response in the 95% of acute horizontal infections requires delineating precisely how the immune systems interact with HBV, and we do not have a clear characterization of the HBV-immune dynamic (Guidotti et al., 2015; Seeger and Mason, 2015). The two levels of infectious response, (i) the innate and (ii) the adaptive, recognize and respond in distinct ways to HBV challenges while modulating each other. Their success or failure influences whether the HBV challenges are resolved with an acute infection and immunity, or a chronic condition with numerous collateral complications (Rehermann, 2013). Innate immune activity, as with the natural killer (NK) cells and resident liver macrophages, or the Kupffer cells, and its more generalized recognition of pathogens are proposed either essentially blind to a “stealth” infection of HBV (Chang and Guo, 2015), or are a critical and “exquisite” guardian against successful HBV colonization (Guidotti et al., 2015). Meanwhile, adaptive responses (e.g., CD4+/CD8+ T-Cells) and their specific targeting of HBV are proposed essential for restricting infection to the transient acute while buttressing immunity against future challenges (Chisari et al., 2010).

Evaluating novel treatments is further complicated by observations of evidently disruptive interactions between innate and adaptive immune activity. Uptake mechanisms observed key to HBV invasion, the sodium-taurocholate co-transporting polypeptide or NTCP (Watashi et al., 2014), is a promising target for blocking HBV uptake. However, innate release of interleukin-6 (IL-6) that interferes with HBV replication and downregulates NTCP also blocks apoptotic mechanisms and may even assist HBV progress (Hösel et al., 2009). The HBV cccDNA itself is a natural prime target, and *in vitro* studies with HepaRG cell lines that show interferon-alpha and lymphotoxin-beta stimulus without cytolytic behavior appears promising (Lucifora et al., 2014), but must be advanced with caution. Innate NK cells were observed to perform unexpected termination of adaptive CD8+ T-cells when pharmacologically stimulated to produce interferon family cytokines (Guidotti et al., 2015). Additionally, dependence of chronic or acute manifestations on the sheer number of HBV suggests an optimal invasion population strategy for HBV (Asabe et al., 2009), presumably evading detection but sufficient for colonization. Unfortunately, direct study of the initial HBV-immune dynamic in patients is difficult or simply not possible, hence the attention given to animal and mathematical models (Murray and Goyal, 2015; Cangelosi et al., 2017).

## Spatial Aspects of Liver Function

Notably, excluded from attention to the HBV-immune dynamic are spatial aspects of hepatic function. Hepatic lobe structure is classically divided per oxygen- and nutrient-rich zones in the liver according to proximity with the portal triad and metabolic function (Gebhardt, 1992; Halpern et al., 2017). More interestingly, the spatial densities of the resident hepatic macrophages, such as Kupffer cells, are weighted heavily around the portal triad inlets (Baratta et al., 2009), putatively reflecting their role as protective filters for the steady flow of antigens into the hepatic matrix. Their activation during HBV challenge as noted with the release of IL-6 appears key to the success of the innate immune response, if any, depending on stealthy HBV invasion. Moreover, as chronic HBV leads to fibrosis, adaptive T-cell monitoring of hepatocytes is compromised by defenestration of sinusoidal epithelia, physically blocking access of CD8+ protrusions for surface antigen surveillance and cytolytic response (Guidotti et al., 2015).

Of note are complications for experimental cellular study of the HBV dynamic with isolated, cultured primary human hepatocytes (PHH). Evidently, differentiation of the PHH disappears outside the liver microenvironment as illustrated by their loss of Cyp450 activity and overall morphological changes within days of culture (Zeisberg et al., 2006; Chen et al., 2012; Rowe et al., 2013). Given the timescales for HBV infection dynamics over weeks, this casts a shadow over cell-based experiments. However, promising efforts emerged with novel application of 3D microfluidic environmental apparatus for PHH, that maintain differentiation for 40 days and further express hallmarks of innate immune activations post-HBV infection—notably with physiologically-observed inoculum of HBV (Ortega-Prieto et al., 2018).

NTCPs face the sinusoidal lumen and present opportunities for HBV invasion that may or may not depend on the metabolic roles of hepatocytes in the different zones. Hence the determination of the spatial distribution of NTCP along the sinusoidal axis becomes crucial for spatial aspects of HBV dynamic. Recently the single-cell RNA sequencing (scRNA-seq) technology combined with *in situ* expression measurements of landmark genes has been applied to measure precise mRNA distributions at distinct lobule coordinates (Halpern et al., 2017). It has been found that around 50% of the expressed liver genes are non-randomly spatially zonated (Halpern et al., 2017). However, the mRNA expression for NTCP, SLC10a4, was not reported in that paper. From the SLC genes studied, SLC22a, SCL13a, and SLC1a do not exhibit a heterogeneous distribution, yet SLC13a and SLC 16a show typical spatial gradients along the sinusoidal axis; these mRNA molecules are less expressed in central vein region than the portal triad region.

Given experimental difficulties and these spatial complexities, we thus emphasize the application of mathematical modeling with attention to potential spatial hepatic heterogeneities of SLC10a4. Spatial distributions in terms of the positioning and density of innate immune cells (e.g., Kupffer, NK) near portal triad inlets need much attention in exploring HBV viral dynamics, since insufficient densities of Kupffer cells may contribute to observed “stealthy” HBV colonizations. Various alignments of hepatocytes in different metabolic zones and defenestration of epithelia restricting access for T-cell surveillance with their potential impact on HBV susceptibility invite further investigation.

## Current Models and Future Direction

Mathematical models have been used to study HBV quite extensively (Ciupe et al., 2007; Fatehi Chenar et al., 2018). Such models have revealed virus production levels, virus clearance rates, and half-life of infected cells. Yet, current modeling studies have not been able to aid in the development of successful HBV drug treatments. A reason for this may lie in the fact that almost all models of HBV ignore the spatial aspects of infection—which we have determined to be quite important for designing HBV treatments.

Current spatial models aimed at perfusion studies and pharmacological processing provide some inspiration (Schwen et al., 2016). *In silico* modeling efforts aimed at hepatic drug perfusion demonstrate a subtle impact of different spatial distributions for hepatocyte steatosis over initial phases of treatment (Schwen et al., 2016). By constructing representative sinusoidal regions over the whole liver organ, blood-flow is modeled through sinusoidal spaces and corresponding variations of hepatic metabolic processing with one-dimensional advection equations coupled to systems of ordinary differential equations assigned to individual hepatocytes (Schwen et al., 2016; Cangelosi et al., 2017). In effect, the representative suites of sinusoid models are distributed over the liver tissue with varying physiologically-based pharmacokinetic (PBPK) models—instead of the entire liver organ (Schwen et al., 2016).

However, purely continuum methods would provide only average levels of HBV; chronic and particularly occult levels of HBV are notoriously difficult to detect and may readily fall below such averages (Guo and Guo, 2015). Thus, agent-based methods for resident hepatic cells and probability distributions for HBV as in Murray and Goyal (2015) are suitable and may be aligned in different spatially-distributed “sample” sinusoid regions analogous to Schwen et al. (2016). Differing densities for immune cells (Kupffer, NK) at key spatial locations as well as cell-level transporter distributions (e.g., the NTCP) are natural inclusions for investigation.

**Figure 1:**
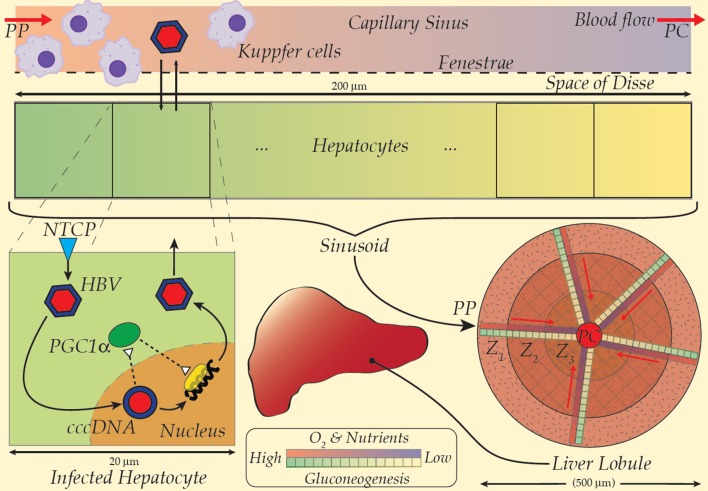
A multiscale model scheme for HBV taking the spatial gradient of NTCP and gluconeogenesis into the model (Refer to the text for details). PP, Peri-portal; PC, Peri-central.

With a continuum model for fluid transport and tissue-level dynamics (e.g., a convection-diffusion equation for the sinusoidal flow) and discrete cell models (e.g., cellular dynamics models of ordinary differential equation systems) over a suite of spatially heterogeneous sample regions, a multi-scaled, detailed and computationally tractable model of the hepatic HBV-immune dynamic is possible (Cangelosi et al., 2017; Franiatte et al., 2019). Low densities of innate immune cells at key spatial locations (e.g., Kupffer near portal-triads), the polarity of cellular distributions of sinusoid-facing transporters (e.g., NTCP), and metabolic roles along the portal-central axis (e.g., hepatic zones I-III) have yet to be investigated for their impact on the HBV infection dynamic. The model in Cangelosi et al. (2017) was interrogated with spatial distributions of NTCP activity, metabolic influence on HBV and a rather simplistic immune response to HBV infection. Nevertheless, substantial spatial heterogeneities remain to be investigated, particularly including above noted immunity response aspects. To improve the current model, the invasion of hepatocytes via NTCP occurs against a backdrop of metabolic and nutrient gradients in the sinusoid may be incorporated (see Figure 1). The essential modulation of blood glucose, particularly via the process of gluconeogenesis, is well-known to operate primarily in Zone 1 or near the periportal (PP) region of the liver lobule (Gebhardt, 1992; Halpern et al., 2017). A protein key to modulation of genes for gluconeogenesis, PGC1-alpha, is evidently targeted and manipulated by HBV for transcription of viral DNA (Bar-Yishay et al., 2011).

Intimately involved in activation of essential transcription factors and their co-activators, PGC1-alpha is the linchpin for hepatocyte and hence liver metabolic nutritional maintenance; its deficient operation cascades through multiple dysfunctions, abnormalities and morbidity (Leone et al., 2005). The link between HBV replication and PGC1-alpha (Shlomai et al., 2006) inspired speculations that HBV manipulation of PGC1-alpha may be central to HBV-induced hepatic steatosis (Bar-Yishay et al., 2011). Nevertheless, the distinctive spatial concentration of gluconeogenesis, its modulation by PGC1-alpha and linkage to HBV replication strongly suggests an intrinsic spatial character to the HBV infection dynamic that has otherwise escaped notice.

Ideally, mathematical modeling techniques for computational tractability from HBV virus, to cell membrane receptors, and to cytokine signaling and cccDNA turnover are naturally required to capture the complex infection process in single-cell models. The single-cell model and its distribution must then be incorporated into a representative sinusoidal system, where the spatial heterogeneity of receptor kick in, and affecting HBV infection distribution over a whole organ. Such a computational pipeline should naturally be capable of taking drug dose input from pharmacokinetic models which target at the whole organism (Kalra et al., 2019)—providing a method for predicting success or failure of such efforts. As spatial distribution effects on HBV replicating, propagation and spreading through inter-cellular spatial arrangements are needed to be studied both in mathematical and animal models. Incorporating spatial aspects of HBV infection into mathematical models has become crucial to study the HBV phenomena. Moreover, sensitivity analysis techniques may assist to assess the sensitivity of parameters on model outputs and fix the parameters for the model, which further may improve the model development and its quality for predictions.

In summary, animal models and *in vitro* experiments for HBV do not provide the level of delicate control over such spatial aspects as provided by mathematical modeling. Construction of such a detailed and, critically, tractable model may prove instrumental in detailing precisely why nearly 300 million people are persistently infected with HBV. Perhaps, also, this vision of a mathematical model for teasing out the delicate HBV dynamics leading to chronic infection can be guided by further experimental efforts. The observed connection between known spatially-distributed enzymes in the liver sinus and HBV replication (Jhuang et al., 2015) is tantalizing. Confirmation of spatial heterogeneities in either simply HBV infection levels or long-term damage to liver tissue would bolster our hypothesis: the complex liver spatial structure is key to HBV infection establishment and may in turn be essential to its elimination.

## Author Contributions

SM, HH, and MA drafted the manuscript. JH attended the conceptual discussion of the paper and reviewed the paper.

### Conflict of Interest

The authors declare that the research was conducted in the absence of any commercial or financial relationships that could be construed as a potential conflict of interest.
